# Xiao'er Xiaoji Zhike Oral Liquid Combined with Azithromycin for *Mycoplasma pneumoniae* Pneumonia in Children: A Systematic Review and Meta-Analysis

**DOI:** 10.1155/2020/9740841

**Published:** 2020-07-19

**Authors:** Gui-Min Zhang, Zhi-Yan Huang, Rong Sun, Shi-Li Ye, Qun Feng

**Affiliations:** ^1^Lunan Hope Pharmaceutical Co., Ltd., Linyi 276006, China; ^2^Lunan Pharmaceutical Group Co., Ltd., State Key Laboratory of Generic Manufacture Technology of Chinese Traditional Medicine, Linyi 276006, China; ^3^National Engineering and Technology Research Centre of Chirality Pharmaceutical, Linyi 276006, China; ^4^The Second Hospital of Shandong University, Ji'nan 250033, China

## Abstract

**Background:**

This study was aimed at systematically evaluating the clinical effect and safety of Xiao'er Xiaoji Zhike oral liquid in the treatment of *Mycoplasma pneumoniae* pneumonia (MPP) in children and providing evidence-based references for clinical application.

**Methods:**

The databases like Chinese Biomedical Literature Database, China Network Knowledge Infrastructure, Wan Fang Database, Chinese Scientific Journal Database, PubMed, EmBase, and the Cochrane Library were systematically investigated via searching clinical trials about Xiao'er Xiaoji Zhike oral liquid in treating MPP from the establishment of these databases to Jun 8, 2020, the valid data from which were entered meta-analysis. The quality of evidence was assessed by GRADE criteria.

**Results:**

Totally, 15 trials and 1500 patients were involved in this review. It showed that clinical efficacy of trial group was more superior than control group at the outcome measures of cough disappearance time, lung rale disappearance time, fever subsidence time, total effective rate, lung X-ray infiltrates disappearing time, reduction of hospital stay, immunological indexes, and some other measures. And the differences between groups were statistically significant. There was no statistical difference in the adverse effects between two groups. Lung X-ray infiltrates disappearing time and cough disappearance time were separately high- and moderate-quality evidences while lung rale disappearance time and fever subsidence time were all low in accordance with GRADE criteria.

**Conclusions:**

In accordance with trials with low methodological quality, Xiao'er Xiaoji Zhike oral liquid combined with azithromycin seems to be safe and superior to azithromycin alone for the treatment of MPP in children. However, further trials with rigorous methodology need to be implemented for these potential benefits.

## 1. Introduction

*Mycoplasma pneumoniae* pneumonia (MPP), also known as primary atypical pneumonia or Eaton's pneumonia, is a kind of community acquired pneumonia (CAP) and up to 40% in CAP. It is a frequently occurring disease in paediatric clinic and its incidence shows an upgrade trend. MPP is a disease where *Mycoplasma pneumoniae* infection leads to respiratory tract infection and the pathological changes in the lung are that of interstitial pneumonia and capillary bronchitis [1, 2]. MPP morbidity seasons take winter and spring as many, but throughout the year obviously. School-age children are the usual victims, and fever, cough, and lung rale are the main clinical features because autoimmune system of school-age children is still not fully developed, which means their immune system against *Mycoplasma pneumoniae* is so weak that these children are susceptible to infection. MPP can go a long time and severely weaken children's health. So, it is easy to induce the injury of many kinds of extrapulmonary organs if interventions are not carried out into MPP children timely and effectively. [3] As a consequence, exploring a safe and effective therapeutic method plays an important role in treating MPP.

At present, macrolides antibiotics are the first choice for the treatment of MPP in clinical practice. As the 2^nd^ generation macrolide antibiotics, azithromycin has the advantage of long half-life period, strong inhibition to mycoplasma, small hepatorenal function damage, and small stomach irritation. In addition, the adjuvant therapy like glucocorticoid, immunoglobulin, and microelement and integrated Chinese-western therapy show a definite effect on MPP in children. [4] However, with the increasing of drug resistance, the proportion of *Mycoplasma pneumoniae* infection gradually rises.

There were reports about traditional Chinese medicine for MPP. Researchers initiate treatment combining macrolides antibiotics on basis of syndrome and its periods and severity. The therapy of combination of Chinese traditional and western medicine is economic, safe, and effective. Xiao'er Xiaoji Zhike oral liquid, as a kind of Chinese patent medicine, refers to a combination of 10 crude drugs and applies to the treatment of pneumonia, cough, dyspepsia, and other diseases in children. Here in the current meta-analysis, we hoped to assess the efficacy and safety of Xiao'er Xiaoji Zhike oral liquid as mono or adjunctive therapy in patients with MPP.

## 2. Materials and Methods

### 2.1. Search Strategy

Four Chinese language electronic databases, namely, Chinese Biomedical Literature Database (Sino-Med), China Network Knowledge Infrastructure (CKNI), Wan Fang Database (WF), and Chinese Scientific Journal Database (VIP), and three English databases (PubMed, EmBase, and the Cochrane Library) were comprehensively searched from inception of each database to June 8, 2020. The following search terms were used individually or combined: pneumonia mycoplasma, primary atypical pneumonia, mycoplasma pneumonia, mycoplasma *pneumoniae* infection, mycoplasma pneumonia infection, azithromycin, Xiao'er Xiaoji Zhike oral liquid, Xiaoerxiaojizhike, et al. All the references and records included were searched to find any additional articles. And all literature reviews were searched and conducted separately by two reviewers according to inclusion and exclusion criteria. When a disagreement came out, the third one was involved until an agreement was reached. NoteExpress software was used for references of duplicate checking and filtering.

### 2.2. Inclusion Criteria

Regarding types of studies design, all included studies in the present systematic review about the treatment of Xiao'er Xiaoji Zhike oral liquid for MPP in children were randomized controlled trials (RCTs), regardless of the methods of blinding, while the languages were set a limit to Chinese and English.

Regarding types of participants, children within 15 years old met the clinical diagnostic criteria for MPP. There was no limitation to the race, gender, or the intensity and course of the disease.

Regarding types of intervention, children in control group were treated with azithromycin and symptomatic treatment of relieving cough and asthma, correcting water and electrolyte balance, keeping breath flowing, or oxygen as needed. Oral azithromycin of 10 mg/kg with a single oral taking on first day (max dosage ≤ 0.5 g) and 5 mg/kg from the second day (max dosage ≤ 0.25 g), tid and intravenous azithromycin of 10 mg/kg by intravenous drip at least 3 days, qd were given, while trial group was treated with the same methods as control group and Xiao'er Xiaoji Zhike oral liquid at the same time. Xiao'er Xiaoji Zhike oral liquid was given with a dosage according to the age, 5 ml within 1 year old, 10 ml between 1 and 2, 15 ml between 2 and 3, and 20 ml over 5, tid. The restriction on dosage or treatment duration of two groups was ignored.

Regarding types of outcomes, (1) primary outcome measures were cough disappearance time, lung rale disappearance time, and fever subsidence time, (2) secondary outcome measures included total effective rate, hospitalization time, lung X-ray infiltrates disappearing time and immunological indexes, and some other outcome measures, and (3) adverse reaction rate. All studies involved one or more of the above outcome measures.

Regarding evaluation criteria, curative effects of studies were judged by special effect, valid, and invalid. (1) Special effect: temperature returned to normal after treatment, clinical symptoms were complete remission and pneumonitis foci were completely abstracted based on X-ray in the chest. (2) Valid: temperature returned to normal after treatment. Special effect: clinical symptoms were remission and pneumonitis foci were improved based on X-ray in chest. (3) Invalid: all signs and symptoms showed no obvious improvement. Total effective rate = special effect rate + valid rate.

### 2.3. Exclusion Criteria

The exclusion criteria were as follows: (1) the studies were not RCTs; (2) reviews, meta-analysis, meeting abstracts, nonclinical studies, or case reports were included; (3) duplicate studies, retrospective studies, and studies without data or with poor design were included; (4) the data was statistically flawed and studies reported improper outcome measures; (5) Xiao'er Xiaoji Zhike oral liquid was administered as a control.

### 2.4. Quality Appraisal

The risk of bias for each included study was assessed by two reviewers according to the Cochrane Collaboration's risk of bias tool [5]. The random sequence generation, allocation concealment, blinding of participants and personnel, blinding of outcome assessment, incomplete outcome data, selective reporting, and conflict of interest were taken into account for the assessment. Disagreements about the risk of assessment were resolved through a discussion with a third reviewer (Shi-Li Ye).

### 2.5. Data Synthesis

Information of studies included was independently extracted by two authors (Qun Feng and Zhi-yan Huang). The detailed information included the title, authors, year of publication, trial design, sample size, baseline characteristics of participants, length of follow-up, method for statistical analysis, inclusion/exclusion criteria, interventions, treatment duration, outcome measures, treatment completed, withdrawal/drop-out, and loss to follow-up [6].

Data analysis was performed using the Review Manager 5.3 software. Forest plot of some outcome measure was used for meta-analysis if the number of studies reached three or more. Otherwise, the measure was assessed by systematic review. Relative risk (RR) was calculated for dichotomous variable, while continuous outcome measures were presented as the weighted mean difference (WMD or MD) and with a 95% confidential interval (CI) rate. Statistical heterogeneity was assessed according to the Cochrane Handbook of Systematic Review of Interventions (version 5.1.0). The heterogeneity was tested by Chi-square and *I*^2^ tests. If there was heterogeneity between the involved studies (*I*^2^ > 50%, *P* < 0.05), the causes of heterogeneity such as gender, age, course and severity of disease, dosage and duration of treatment, and subgroup analysis were searched for. If the cause was not found, the random-effect model for meta-analysis was used. Subgroup analysis or sensitivity analysis was performed to eliminate heterogeneity based on possible heterogeneous factors. [7] The fixed-effect model was applied if there was no heterogeneity between the studies. Publication bias was assessed by using a funnel plot when there were more than 10 trials in the meta-analysis. Publication bias was also examined by test of Egger, test of Begg, and Luis Furuya-Kanamori (LFK) index [8–10]. In addition, two reviewers used the GRADE profiler 3.6 software to rate the quality of evidence. Disagreements were still resolved through a discussion with a third reviewer.

## 3. Results

### 3.1. Characteristics of Studies

**Figure 1:**
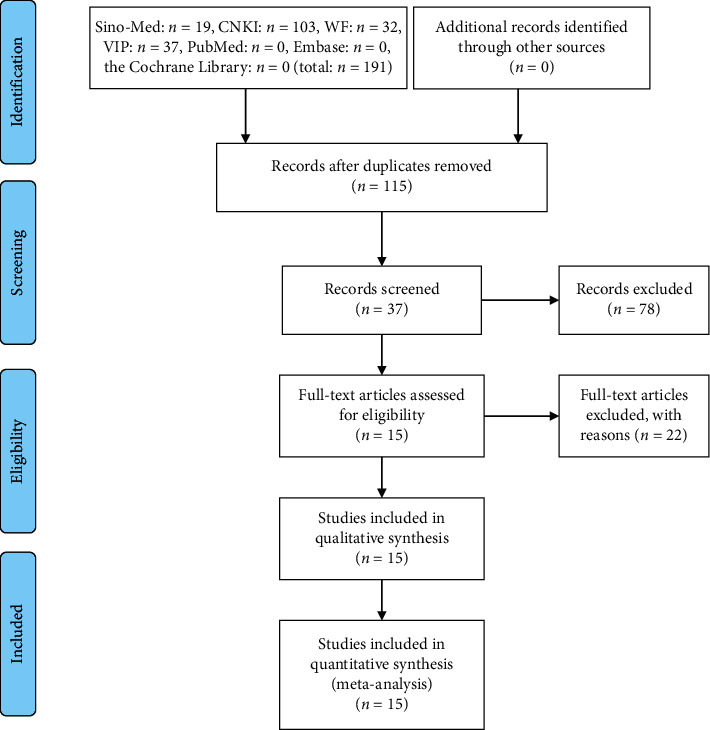
Flow chart of study selection.

A total of 191 articles were comprehensively investigated according to the previous search strategy. Among 15 articles reported in Chinese involving 1500 children with MPP, 752 cases in trial group and 748 in control group had undergone meta-analysis [11–25]. The screening process and reasons of exclusion are summarized in Figure 1 and the basic characteristics of the included trials are detailed in Table 1.

### 3.2. Quality Assessment

**Figure 2:**
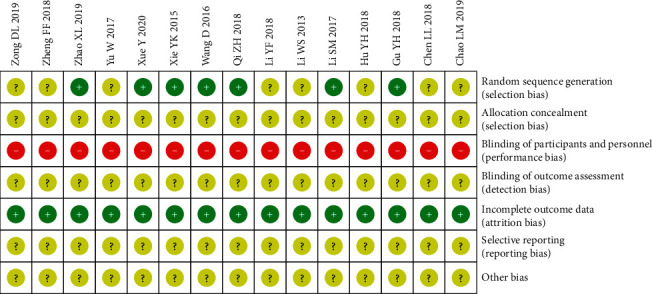
Risk of bias summary.

All the studies included were assessed to be low methodological quality according to the Cochrane Collaboration's risk of bias tool. Random sequence generation method was used in seven articles. Two articles used the ballot [14, 23] and five used the numbers method [12, 15, 16, 18, 20], while the remaining other trials did not provide the detailed information. Allocation concealment, blinding of outcome assessment, selective reporting, and other bias were not conducted in all trials. Every study did not detail the blinding of participants and personnel. But the method could be broken. There were no trials having incomplete outcome data (Figure 2).

### 3.3. Outcome Measures

Azithromycin was used with oral administration in eight trials [11–18] and intravenous injection in seven [19–25]. Herein, this review analysed all outcome measures with subgroup analysis based on the different ways of administration.

#### 3.3.1. Cough Disappearance Time

**Figure 3:**
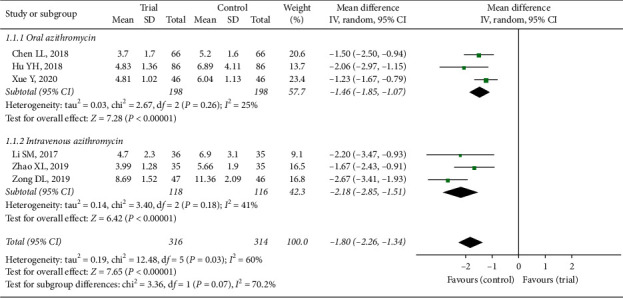
Forest plot of cough disappearance time.

Six articles [11, 13, 18, 20, 23, 25] paralleled the efficacy of shortening cough time between trial group and control group. Six studies were totally analysed and the results between two groups (MD = −1.80, 95% CI −2.26 to −1.34) in Figure 3 exhibited significant difference in cough disappearance time (*Z* = 7.65, *P* < 0.00001) based on random-effect model (*P*=0.03, *I*^*2*^ = 60%). Three trials exerted azithromycin orally comparing to drugs combination (MD = −1.46, 95% CI: −1.85 to −1.07, *Z* = 7.28, *P* < 0.00001). Azithromycin with intravenous injection was applied in another three articles (MD = −2.18, 95% CI: −2.85 to −1.51, *Z* = 6.42, *P* < 0.00001). Heterogeneity between two subgroups (*P*=0.07, *I*^2^ = 70.2%) might result from the usage of azithromycin. The results revealed that compared with applying azithromycin alone, cough time of children with Xiao'er Xiaoji Zhike oral liquid and azithromycin was nearly 2 days shorter, which showed great clinical value.

#### 3.3.2. Lung Rale Disappearance Time

**Figure 4:**
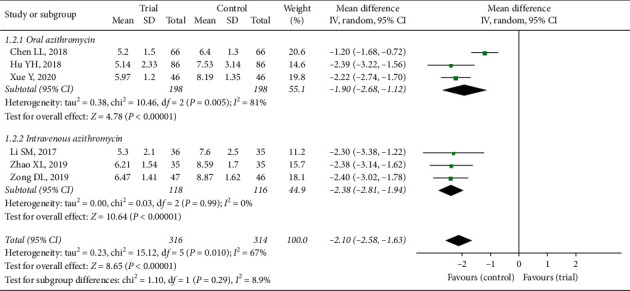
Forest plot of lung rale disappearance time.

In this review, six articles [11, 13, 18, 20, 23, 25] showed the efficacy of shortening lung rale time between two groups. Three trials used azithromycin orally comparing to drugs combination (MD = −1.90, 95% CI: −2.68 to −1.12, *Z* = 4.78, *P* < 0.00001) based on random-effect model (*P*=0.005, *I*^2^ = 81%). Azithromycin with intravenous injection was also applied in three trials (MD = −2.38, 95% CI: −2.81 to −1.94, *Z* = 10.64, *P* < 0.00001). Six trials were totally analysed and the results between trial group and control group (MD = −2.10, 95% CI: −2.58 to −1.63) in Figure 4 revealed significant difference in lung rale disappearance time (*P* < 0.00001) according to random-effect model (*P*=0.010, *I*^2^ = 67%). There was heterogeneity, whether between two subgroups or among 6 trials, coming from the difference of test methods and standards [11]. But the result was not reversible, whether the article was included or not. So, the results indicated that compared with applying azithromycin alone, lung rale time of children with the treatment of Xiao'er Xiaoji Zhike oral liquid and azithromycin was more than 2 days shorter, which might be great clinical value.

#### 3.3.3. Fever Subsidence Time

**Figure 5:**
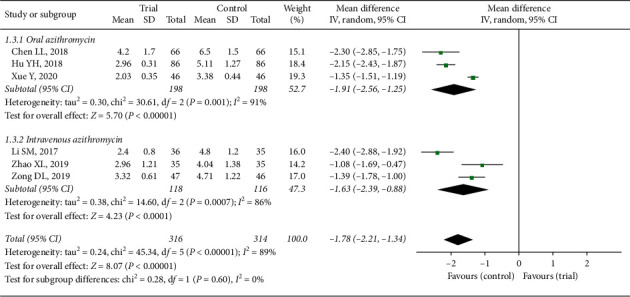
Forest plot of fever subsidence time.

The efficacy of fever subsidence time was reported in six studies [11, 13, 18, 20, 23, 25] in this review. Random-effect model was used in this outcome because of the heterogeneity in each subgroup and the total analysis (*P* < 0.05, *I*^2^ > 50%). Three trials with oral azithromycin were compared with drugs combination (MD = −1.91, 95% CI: −2.56 to −1.25, *Z* = 5.70, *P* < 0.00001) and three were applying azithromycin intravenous injection (MD = −1.63, 95% CI: −2.39 to −0.88, *Z* = 4.23, *P* < 0.0001), while the analysis results of fever subsidence time between two groups (MD = −1.78, 95% CI: −2.21 to −1.34) in Figure 5 illustrated a remarkable difference (*Z* = 8.07, *P* < 0.00001). The reason of heterogeneity might also be assessment methods and standards in different studies. The results indicated that fever time of children with the treatment of Xiao'er Xiaoji Zhike oral liquid and azithromycin was nearly 2 days shorter than that with azithromycin alone.

#### 3.3.4. Total Effective Rate

All the included RCTs compared the clinical total efficacy between trial group and control group. It was assessed with three classes: special effect, valid, and invalid.

**Figure 6:**
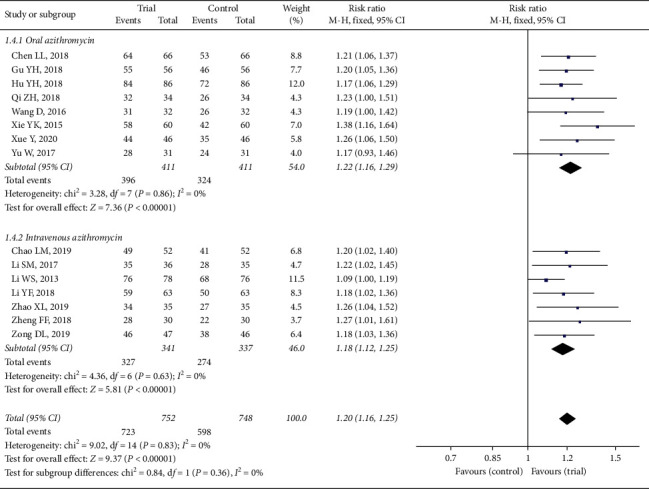
Forest plot of total efficiency based on usage of azithromycin.

There was no heterogeneity in two subgroups or in the total analysis (*P* > 0.05, *I*^2^ = 0). Therefore, a fixed-effect model was taken for the statistical analysis. The analysis of two subgroups (oral azithromycin: RR = 1.22, 95% CI: 1.16 to 1.29, *P* < 0.00001; intravenous azithromycin: RR = 1.18, 95% CI: 1.12 to 1.25, *P* < 0.00001) in Figure 6 exhibited significant difference. Meanwhile, there was no heterogeneity between two subgroups (*P*=0.36, *I*^2^ = 17.9%). The result of all trials (RR = 1.20, 95% CI: 1.16 to 1.25) made it clear that the efficacy with Xiao'er Xiaoji Zhike oral liquid and azithromycin was remarkably better than that with azithromycin alone.

**Figure 7:**
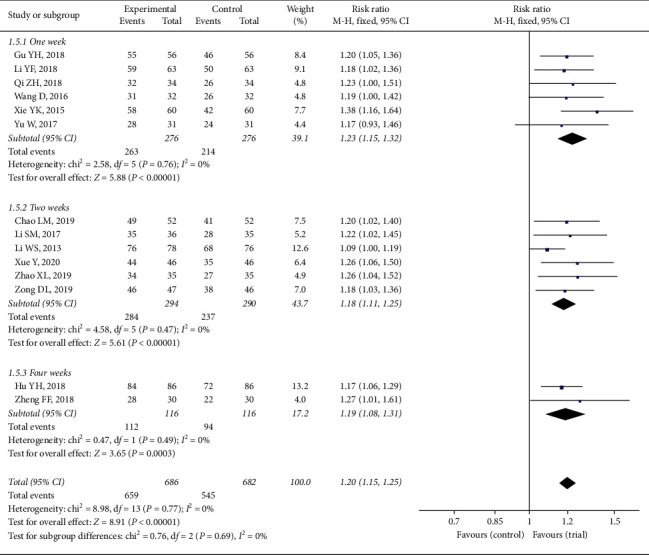
Forest plot of total efficiency based on treatment duration.

In addition, treatment duration (one, two and four weeks) of two groups was also analysed in Figure 7. There was no heterogeneity in three subgroups or in the total analysis (*P* > 0.05, *I*^2^ = 0). There was also no significant difference among the three groups, respectively (*P*=0.69).

#### 3.3.5. Lung X-Ray Infiltrates Disappearing Time

**Figure 8:**
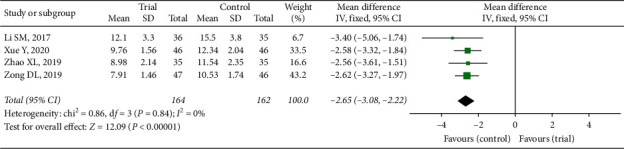
Forest plot of X-ray infiltrates disappearing time.

Four trials mentioned this outcome measure and azithromycin of all were administrated with intravenous injection [18, 20, 23, 25]. The fixed-effect model was used in Figure 8 because of heterogeneity test across these studies (*P*=0.84, *I*^2^ = 0%). The MD was −2.65 (*Z* = 12.09, *P* < 0.00001) with a 95% CI of −3.08 to −2.22. Therefore, two groups showed significant differences in the ability to reduce lung X-ray infiltrates time.

#### 3.3.6. Immunological Indexes

**Figure 9:**
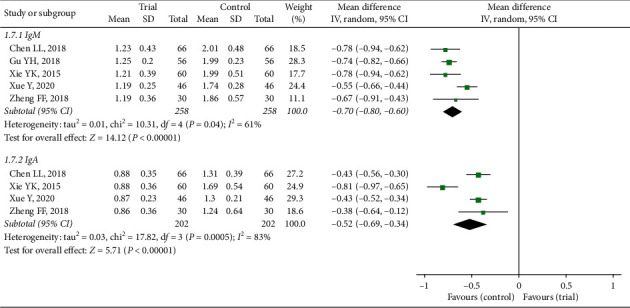
Forest plot of IgM and IgA.

Immunological indexes, reported in five trials altogether, included IgM, IgA, IgG, C3, C4, CD3+, CD4+, and CD4+/CD8+. Two trials mentioned IgG [11, 22], C3, and C4 [11, 16] separately and one [23] mentioned CD3+, CD4+, and CD4+/CD8+. Every index in the studies indicated the statistical effect, respectively. In the forest plot of IgM and IgA (Figure 9), studies were totally analysed and the results between trial group and control group (IgM : MD = −0.70, IgA : MD = −0.52) exhibited significant difference in cough disappearance time (IgM : *Z* = 14.12, *P* < 0.00001; IgA : *Z* = 5.71, *P* < 0.00001) based on random-effect model (IgM : *P* = 0.04, *I*^2^ = 61%; IgA : *P*=0.0005, *I*^2^ = 83%). The trial was the source of heterogeneity in IgM subgroup [18] and another in IgA [16]. However, the results were not reversible whether including the articles or not.

#### 3.3.7. Other Outcome Measures

Two trials [13, 21] mentioned hospitalization time. There was heterogeneity because of the difference of course duration and administration of azithromycin. So, the combined analysis was ignored, regardless of the effect of shortening hospitalization time statistically in each study. Inflammatory factors (IL-6, IL-8, and TNF-*α*) were reported in a trial [19]. These factors in trial group reduced significantly compared with controls after treatment. One trial reported that the level of C-reactive protein after the treatment of Xiao'er Xiaoji Zhike oral liquid and azithromycin was remarkably lower than that with azithromycin alone [22].

### 3.4. Safety Assessment

**Figure 10:**
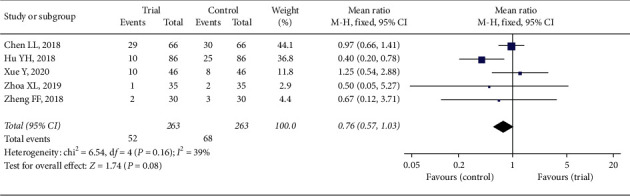
Forest plot of adverse reactions.

Five studies reported adverse events involving 120 children (52 in trial group and 68 in control group) [11, 13, 18, 23, 24]. The adverse reactions with high incidence appeared in Table 2. Besides, there was one child with exciting laryngeal in trial group, one with hoarseness and one with local pain in control group. All reactions were mild and did not affect the therapeutic process. Digestive system reactions had the highest incidence, but there was no significant difference between two groups. All adverse reactions were labelled in the direction of azithromycin and none in the direction of Xiaoji Zhike oral liquid. The results of two groups (RR = 0.76, 95% CI: 0.57 to 1.03) in Figure 10 exhibited no significance in adverse reactions (*Z* = 1.74, *P*=0.08) based on fixed-effect model (*P*=0.16, *I*^2^ = 39%). It indicated that these adverse reactions had no direct connection with taking Xiao'er Xiaoji Zhike oral liquid.

### 3.5. Publication Bias

**Figure 11:**
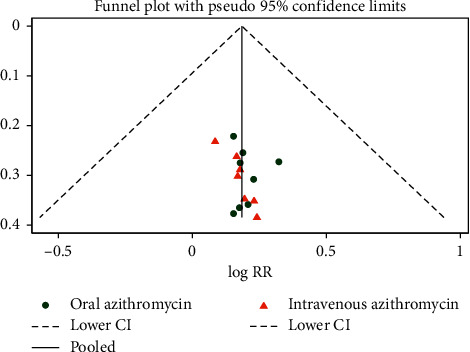
Funnel plot of total efficiency.

**Figure 12:**
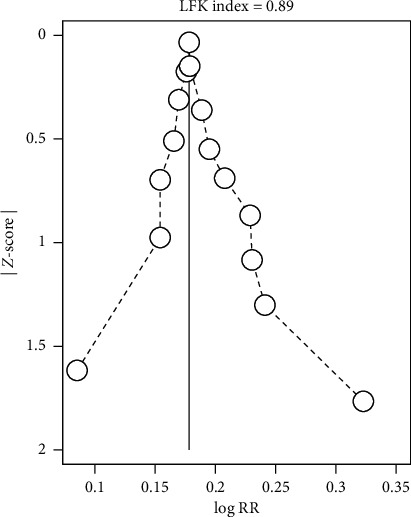
Doi plot of total efficiency.

Approximately symmetric distribution was illustrated according to the funnel plot and Doi plot of total efficiency (Figures 11 and 12) suggesting that there may be no publication bias. There was also no significant indication of publication bias from the test of Egger (*P*=0.172, 95% CI: −0.20 to 1.02), Begg (*Z* = 1.29, *P*=0.198), and LFK index (0.89). In aggregate, publication bias does not exist in this review.

### 3.6. Grade Quality of Evidence

The quality of evidences was assessed according to the five factors: risk of bias, inconsistency, indirectness, imprecision, and publication bias. Based on GRADE criteria, Lung X-ray infiltrates disappearing time and cough disappearance time were separately high- and moderate-quality evidences. Lung rale disappearance time and fever subsidence time were all low-quality evidences (Table 3).

## 4. Discussion

Children with MPP usually have the symptoms of cough and expectoration. So not only infection should be resisted with azithromycin, but also some antipyretic, antitussive, and expectorant drugs need to be applied. Azithromycin, a kind of macrolides antibiotics, is used for bacterial infection. Gastrointestinal reaction and anaphylactic reaction are the common adverse reactions. Oral azithromycin is safer than intravenous azithromycin, but lower of bioavailability than the latter. Xiao'er Xiaoji Zhike oral liquid, as a kind of Chinese patent medicine and widely used for digestive and respiratory diseases, has antipyretic, antitussive, and expectorant effect and shows remarkable results.

This review pays attention to evaluating the efficacy and safety of Xiao'er Xiaoji Zhike oral liquid in combination with oral or intravenous azithromycin for treating MPP in children compared with azithromycin alone. Through the strict selecting, 15 RCTs meet the criteria, including 1500 paediatric patients with MPP. But all of them are single-centre RCTs with small sample size, which range from 60 to 172. Most of the trials are considered to have a high risk of bias. Because few of them have designed or detailed a complete protocol for the randomized controlled trials, only seven articles have described generation methods of random sequence. None of them have used or described allocation concealment and blinding, which may more likely subjectively exaggerate the treatment effects of trial group. Nonetheless, we detect no significant publication bias in this review by funnel plot, Begg's test, Egger's test, and LFK index.

The data are extracted for systematic review according to evaluation of outcomes, involving total effective rate, cough disappearance time, lung rale disappearance time, fever subsidence time, lung X-ray infiltrates disappearing time, immunoglobulins, adverse events, and so on. All of them focused on the effective rate of drugs on MPP, but only six trials referred to the primary outcome measures. The results revealed that Xiao'er Xiaoji Zhike oral liquid, serving as the therapy for MPP, functioned with higher efficacy in terms of decreasing cough disappearance time, lung rale disappearance time, fever subsidence time, increasing the total effective rate, and improving other outcome measures than that of control group, regardless of the numbers of trials each outcome included. According to the subgroup analysis of the total effective rate, there is no significant difference between subgroups about the usage of azithromycin and the same to the comparison of treatment course respectively among one week, two weeks, and four weeks. The treatment course is determined by the age, habitus, onset time, and severity. All included trials just have detected the final state, which results in the absence of difference regardless of treatment durations. Moreover, the effects of trial group are all better than that of control group in every subgroup. So, based on the performed systematic review and meta-analysis, the combination therapy appears to be superior to azithromycin alone. Only five included trials mentioned the presence and absence of adverse reactions. Although the combined application of drugs does not increase the incidence of adverse reactions, we cannot make an absolutely accurate decision.

The quality of evidences reflects the professional power we have owned to sustain some specific recommendation, which provides a reference for clinic [26]. Based on the GRADE criteria, the quality of evidences but lung X-ray infiltrates disappearing time and cough disappearance time is unsatisfactory. The conclusions may be attributable to trials' lack in quantity and quality and the difference of checking and assessment.

Meanwhile, limitations in this review may exist, which give us clear indicators for the further studies. First, the quality of all includes trials that need improvement from the aspect of RCT methodology and statistics. The Cochrane Handbook guides how researchers design a high-quality RCT from the aspects of comparable baseline, comprehensive outcome measures, follow-up, statistics, and so on. Second, lack of multicentre studies with large sample may cause unreliable assessments of Xiao'er Xiaoji Zhike oral liquid. Furthermore, only the Chinese and English databases were searched in view of the fact that Xiao'er Xiaoji Zhike oral liquid was a kind of Chinese patent medicine mainly applied in China, which may reduce the test efficiency and cause some selection bias.

## 5. Conclusions

This review preliminarily indicates that Xiao'er Xiaoji Zhike oral liquid combined with azithromycin is quite effective and safe for children with MPP compared with the treatment of azithromycin alone. However, more double-blind and multicentre RCTs with high quality, large sample, and adequate follow-up is required for higher evidence-based therapies in the future.

## Figures and Tables

**Table 1:** Basic characteristics of included studies.

Study		Size T/C	Sex male/female	Age		Interventions		Course duration	Outcome measures
				T	C	T	C		
Chen [11]		66/66	69/63	6.5 ± 2.4	6.4 ± 2.5	XXZ + Azithromycin, p.o.	Azithromycin, p.o.	10 days	①②③④⑥⑧
Gu [12]		56/56	84/28	7.1 ± 1.0	6.8 ± 1.1	XXZ + Azithromycin, p.o.	Azithromycin, p.o.	1 week	④⑥
Hu [13]		86/86	83/89	8.25 ± 3.71	8.31 ± 2.55	XXZ + Azithromycin, p.o.	Azithromycin, p.o.	4 weeks	①②③④⑦⑧
Qi [14]		34/34	42/26	4.6 ± 1.9	4.8 ± 2.2	XXZ + Azithromycin, p.o.	Azithromycin, p.o.	1 week	④
Wang [15]		32/32	31/33	7.54 ± 2.75	6.32 ± 2.14	XXZ + Azithromycin, p.o.	Azithromycin, p.o.	1 week	④
Xie [16]		60/60	61/59	5.19 ± 2.38	5.21 ± 2.46	XXZ + Azithromycin, p.o.	Azithromycin, p.o.	1 week	④⑥
Yu [17]		31/31	30/32	6.32 ± 2.12	6.94 ± 2.76	XXZ + Azithromycin, p.o.	Azithromycin, p.o.	1 week	④
Xue [18]		46/46	55/37	5.41 ± 1.10	5.38 ± 1.25	XXZ + Azithromycin, p.o.	Azithromycin, p.o.	2 weeks	①②③④⑤⑥⑧
Chao [19]		52/52	62/42	5.27 ± 1.32	5.13 ± 1.26	XXZ + Azithromycin, i.v.	Azithromycin, i.v.	2 weeks	④⑦
Li [20]		36/35	39/32	6.6 ± 2.5	6.2 ± 2.1	XXZ + Azithromycin, i.v.	Azithromycin, i.v.	2 weeks	①②③④⑤
Li [21]		78/76	82/72	5.6 ± 1.3	5.5 ± 1.2	XXZ + Azithromycin, i.v.	Azithromycin, i.v.	2 weeks	④⑦
Li and Jin [22]		63/63	70/56	5.6 ± 1.3	5.5 ± 1.2	XXZ + Azithromycin, i.v.	Azithromycin, i.v.	1 week	④⑦
Zhao [23]		35/35	39/31	7.55 ± 2.06	7.51 ± 2.02	XXZ + Azithromycin, i.v.	Azithromycin, i.v.	2 weeks	①②③④⑤⑧
Zheng et al. [24]		30/30	31/29	8.01 ± 2.32	8.47 ± 2.06	XXZ + Azithromycin, i.v.	Azithromycin, i.v.	30 days	④⑥⑧
Zong [25]		47/46	51/42	3.27 ± 0.77	3.19 ± 0.68	XXZ + Azithromycin, i.v.	Azithromycin, i.v.	15 days	①②③④⑤

XXZ, Xiao'er Xiaoji Zhike oral liquid; T, trial group; C, control group; ①cough disappearance time, ②lung rale disappearance time, ③fever subsidence time, ④total effective rate, ⑤lung X-ray infiltrates disappearing time, ⑥immunological indexes, ⑦others, and ⑧adverse reaction.

**Table 2:** Adverse events of included studies.

Author, year	Size (T/C)	Nausea and vomiting (T/C)	Constipation (T/C)	Gastrointestinal discomfort (T/C)	Headache (T/C)	Allergic reaction (T/C)
Chen, 2018 [11]	66/66	3/2	13/14	10/11	1/2	2/1
Xue, 2020 [18]	46/46	—	—	4/3	0/1	2/2
Zhao, 2019 [23]	35/35	—	—	1/1	4/3	—
Zheng, 2018 [24]	30/30	—	—	2/2	—	—

**Table 3:** The grade quality of evidence.

Xiao'er Xiaoji Zhike oral liquid and azithromycin compared to azithromycin for MPP in children						
Patient or population: patients with MPP Intervention: Xiao'er Xiaoji Zhike oral liquid and azithromycin Comparison: azithromycin						

Outcomes	Illustrative comparative risks^*∗*^ (95% CI)		Relative effect (95% CI)	Number of participants (studies)	Quality of the evidence (GRADE)	Comments
	Assumed risk	Corresponding risk				
	Azithromycin	Xiao'er Xiaoji Zhike oral liquid and azithromycin				

Cough disappearance time Scale from 0 to 2	The mean cough disappearance time ranged across control groups from 3.70 to 11.36 d	The mean cough disappearance time in the intervention groups was 1.80 lower (2.26 to 1.34 lower)		630 (6 studies)	⊕ ⊕ ⊕ ⊝ Moderate^1,2^	

Lung rale disappearance time Scale from 0 to 2	The mean lung rale disappearance time ranged across control groups from 5.14 to 8.87 d	The mean lung rale disappearance time in the intervention groups was 2.10 lower (2.58 to 1.63 lower)		630 (6 studies)	⊕ ⊕ ⊝ ⊝ Low^1,2,3^	

Fever subsidence time Scale from 0 to 1	The mean fever subsidence time ranged across control groups from 2.03 to 6.50 d	The mean fever subsidence time in the intervention groups was 1.78 lower (2.21 to 1.34 lower)		630 (6 studies)	⊕ ⊕ ⊝ ⊝ Low^1,2,3^	

Lung X-ray infiltrates disappearing time Scale from 0 to 3	The mean lung X-ray infiltrates disappearing time ranged across control groups from 7.91 to 15.50 d	The mean lung X-ray infiltrates disappearing time in the intervention groups was 2.65 lower (3.08 to 2.22 lower)		326 (4 studies)	⊕ ⊕ ⊕ ⊕ High	

^*∗*^The basis for the assumed risk (e.g., the median control group risk across studies) is provided in footnotes. The corresponding risk (and its 95% confidence interval) is based on the assumed risk in the comparison group and the relative effect of the intervention (and its 95% CI). CI : confidence interval.						

GRADE working group grades of evidence. High quality: further research is very unlikely to change our confidence in the estimate of effect. Moderate quality: further research is likely to have an important impact on our confidence in the estimate of effect and may change the estimate. Low quality: further research is very likely to have an important impact on our confidence in the estimate of effect and is likely to change the estimate. Very low quality: we are very uncertain about the estimate.						

^1^Three trials did not detail the random sequence generation method. ^2^Allocation concealment, blinding of outcome assessment, selective reporting, and other bias were not conducted in all trials. ^3^Heterogeneity is obvious based on *I*^2^ being more than 70%.

## Data Availability

All data analysed or generated during this work are included within the article.
